# Optical-cavity mode squeezing by free electrons

**DOI:** 10.1515/nanoph-2022-0481

**Published:** 2022-10-10

**Authors:** Valerio Di Giulio, F. Javier García de Abajo

**Affiliations:** ICFO-Institut de Ciencies Fotoniques, The Barcelona Institute of Science and Technology, 08860 Castelldefels, Barcelona, Spain; ICREA-Institució Catalana de Recerca i Estudis Avançats, Passeig Lluís Companys 23, 08010,Barcelona, Spain

**Keywords:** light–matter interaction, ponderomotive interaction, quantum optics, squeezed states

## Abstract

The generation of nonclassical light states bears a paramount importance in quantum optics and is largely relying on the interaction between intense laser pulses and nonlinear media. Recently, electron beams, such as those used in ultrafast electron microscopy to retrieve information from a specimen, have been proposed as a tool to manipulate both bright and dark confined optical excitations, inducing semiclassical states of light that range from coherent to thermal mixtures. Here, we show that the ponderomotive contribution to the electron–cavity interaction, which we argue to be significant for low-energy electrons subject to strongly confined near-fields, can actually create a more general set of optical states, including coherent and squeezed states. The postinteraction electron spectrum further reveals signatures of the nontrivial role played by *A* ^2^ terms in the light–matter coupling Hamiltonian, particularly when the cavity is previously excited by either chaotic or coherent illumination. Our work introduces a disruptive approach to the creation of nontrivial quantum cavity states for quantum information and optics applications, while it suggests unexplored possibilities for electron beam shaping.

## 1 Introduction

The generation of different states of light is of fundamental interest in quantum optics and enables powerful applications such as the increase in sensitivity achieved in the interferometric detection of gravitational waves through the use of squeezed states with reduced uncertainty [1]. Likewise, the generation of approximate Gottesman–Kitaev–Preskill (GKB) states [2] is needed to implement fault-tolerant quantum computing on photonic setups [3]. In this context, quantum two-photon states are commonly produced by exploiting the nonlinear response of some materials to coherent laser illumination through processes such as four-wave mixing [4] and parametric down-conversion [5] assisted by bright modes in optical cavities. In a radically different approach, free electrons have been identified as a promising tool to generate quantum light states [6, 7]. In addition to the nanometer precision with which electron beams (e-beams) can be focused on an optical cavity, they are advantageous with respect to external light excitation in that they can interact with strongly confined dark modes because they act as broad sources of evanescent fields [8].

The interest in generating low-uncertainty states through e-beams dates back to the early stages in the development of free-electron lasers, when several theoretical works predicted this kind of statistics in the free-space radiation emission from a linear wiggler [9–11]. More recently, with the advent of ultrafast electron microscopy [12–14], the experimental ability of integrating intense laser sources and e-beams in a single setup allowed for their synchronized interaction at the specimen, giving rise to the so-called photon-induced near-field electron microscopy (PINEM) [15]. In this technique, the strong inelastic electron coupling to optical fields scattered by the specimen results in the absorption and emission of multiple light quanta, causing a substantial reshaping of the electron wave function as well as the state of the cavity modes targeted by the laser at the specimen.

From the point of view of the electron, such reshaping consists in the emergence of intense sidebands in the electron spectrum spaced by the laser photon energy [16]. Interestingly, upon further propagation over macroscopic distances, coherent electron components possessing different energies (and velocities) evolve into a train of attosecond pulses [17–19]. In addition, our ability to manipulate the electron density matrix can be extended by using squeezed light instead of coherent laser sources [20].

From the perspective of an optical mode in the specimen, the electron is known to simply act as a displacement operator on the coherent state produced by the external illumination [21]. In particular, the electron–cavity interaction leads to a density matrix with Poissonian diagonal elements when the cavity is initially prepared in the ground state [20]. Although more complicated states can be obtained from consecutive interactions with multiple electrons combined with projective measurements onto specific electron states [6, 22, 23], single electrons have so far been regarded as a semiclassical current acting on the specimen. This is indeed a robust assumption for energetic electrons whose velocity **v** is negligibly perturbed by the interaction (nonrecoil approximation), provided linear terms in the electron–light coupling Hamiltonian (i.e., ∝ **v** · **A**, where **A** is the vector potential) are dominant over ponderomotive ∝ *A* ^2^ contributions. This condition is, however, challenged for an electric field associated with a mode polarized normally to the electron propagation direction, or when sign cancellations render a vanishing linear coupling coefficient, as well as when low-energy electrons such as those used in low-energy electron microscopy (LEEM) [24, 25] (≲100 eV) are made to interact with cavities supporting resonances in the infrared range. In fact, when linear and quadratic **A** terms have similar strengths in the coupling Hamiltonian, we expect the effect of a single electron on a cavity mode to deviate from the classical-current description, leading, for instance, to the direct production of squeezed cavity states.

**Figure 1:**
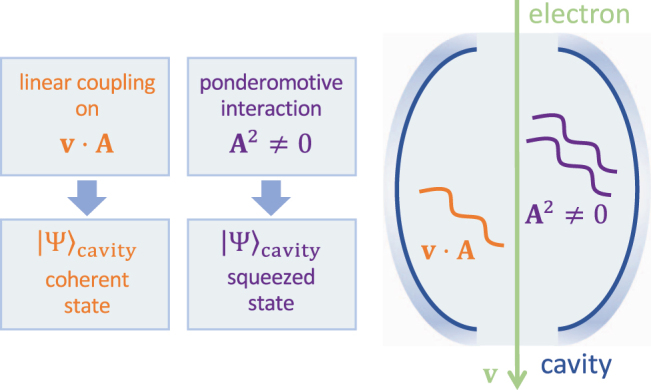
Free-electron interaction with an optical cavity. We present a sketch of the interaction for a single-mode cavity. Both linear (∝ **v** · **A**) and quadratic (ponderomotive ∝ *A* ^2^) terms in the quantum vector potential operator **A** are present in the minimal-coupling light–matter interaction Hamiltonian. Switching on and off these two terms selects the creation of either coherent or squeezed cavity mode states, respectively.

In this work, we study the interaction of a free electron with a single-mode cavity under conditions in which linear and ponderomotive interactions have commensurate strengths or when the latter dominates (Figure 1). We start by developing the theory describing the interaction of a fast electron with a single-mode cavity in the nonrecoil approximation including the effect of the quantized ponderomotive coupling, thus allowing us to study the interaction with poorly populated cavities and compute observables connected with the mode. This is in contrast to previous works that incorporated a classical description of the vector potential and, therefore, implicitly assumed that the cavity was prepared in a high-fluence coherent state [26–28]. Remarkably, the entire dynamics admits an analytical solution in terms of the consecutive action of a displacement operator and a squeezing operator. Then, we theoretically demonstrate that a cavity starting from its ground state is left in a general two-photon coherent state [29] after interaction with a single electron, including vacuum-, phase-, and amplitude-squeezed states. Finally, we study how the presence of the ponderomotive interaction can lead to asymmetric distributions in the electron spectrum both when the cavity is initially heated (i.e., prepared in a thermal state) or when it is irradiated by laser light (i.e., in an initial coherent state). Besides their relevance from a fundamental viewpoint, our results suggest a way to produce nonclassical states of light in a given dark mode that is accessible to electrons by coupling to the evanescent components of their accompanying electric fields.

## 2 Results and discussion

### 2.1 Electron–cavity quantum dynamics

#### 2.1.1 The evolution operator

Following the methods introduced in Ref. [21], we start by considering a structure characterized by a single photonic mode of energy *ℏω*_0_ satisfying a bosonic statistics and interacting with an incoming electron initially prepared with a wave function $\psi_{0}\left(r,t\right)=e^{i\left(E_{0}t/\hbar -k_{0}\cdot r\right)}\phi_{0}\left(r-vt\right)$ . This expression assumes that *ψ*_0_(**r**, *t*) is made of momentum components that are closely peaked around a value *ℏ* **k**_0_ corresponding to a central energy $E_{0}=c\sqrt{m_{e}^{2}c^{2}+\hbar^{2}k_{0}^{2}}$ , such that the function *ϕ*_0_(**r** − **v** *t*) varies slowly in space and time. By further assuming the lifetime of the photonic mode to be much longer than the electron–specimen interaction time, and adopting the nonrecoil approximation, the dynamics of the combined electron–cavity system can be well described by the Schrödinger equation $i\hbar \partial_{t}|\psi \left(r,t\right)〉=\left({\hat{H}}_{0}+{\hat{H}}_{\text{int}}\right)|\psi \left(r,t\right)〉$ with free and interaction Hamiltonians given by (see Methods, Section 4.1.1)

(1a) $${\hat{H}}_{0}=\hbar \omega_{0}a^{†}a+E_{0}-\hbar v\cdot \left(i\nabla +k_{0}\right),$$

(1b) $${\hat{H}}_{\text{int}}=\left(ev/c\right)\cdot \hat{A}\left(r\right)+\underset{i}{\sum }g_{i}{\hat{A}}_{i}^{2}\left(r\right),$$

where $\hat{A}\left(r\right)=\left(-ic/\omega_{0}\right)\left[{\vec{E}}_{0}\left(r\right)a-{\vec{E}}_{0}^{*}\left(r\right)a^{†}\right]$ is the quantized vector potential operator incorporating the normalized electric field mode profile ${\vec{E}}_{0}\left(r\right)$ , the sum over *i* runs over Cartesian components, and the vector **g** = (*e* ^2^/2*m*_e_ *c* ^2^ *γ*)(1, 1, *γ* ^−2^) with $\gamma =1/\sqrt{1-{\left(v/c\right)}^{2}}$ introduces approximate relativistic corrections in the ponderomotive interaction. We remark that the nonrecoil approximation that we adopt to describe the electron–cavity interaction is reasonable provided the condition *ℓℏω*_0_/*E*_0_ ≪ 1 is satisfied (e.g., for keV electrons and cavities resonating at visible or lower frequencies). More precisely, as shown in Ref. [30], the solution including recoil already converges to the nonrecoil limit for *ℓℏω*_0_/*E*_0_ ∼ 0.3. For example, it is safe to ignore recoil for interaction strengths leading to *ℓ* ∼ 25, using *E*_0_ ∼ 5 eV electrons that couple to a *ℏω*_0_ ∼ 60 meV cavity.

As explained in detail in Methods, Section 4.1.2, given the initial condition $|\psi \left(r,t\to -\infty \right)〉=\psi_{0}\left(r,t\right)\sum_{n}\alpha_{n}^{0}e^{-in\omega_{0}t}|n〉$ (i.e., uncorrelated electron and cavity states), the problem admits an analytical solution that represents a generalization to the one found in Ref. [21], including the effect of the single-mode ponderomotive force [the *A* ^2^ terms in Eq. (15b)]. More precisely, taking the electron velocity as $v=v\hat{z}$ , we find

(2) $$|\psi \left(r,t\right)〉=\psi_{0}\left(r,t\right)\underset{\ell ,n}{\sum }e^{i\omega_{0}\left[\ell \left(z/v-t\right)-nt\right]}e^{i\theta_{n}\left(r\right)}f_{\ell }^{n}\left(r\right)|n〉,$$

where the phases $\theta_{n}=-\sum_{i}\int_{-\infty }^{z}dz^{\prime }|\eta_{i}\left(z^{\prime }\right){|}^{2}\left(2n+1\right)$ incorporate a dependence on the mode field and electron velocity through

(3) $$\eta_{i}\left(z\right)=\sqrt{\frac{g_{i}c^{2}}{v\hbar \omega_{0}^{2}}}\;E_{0,i}\left(z\right)e^{-i\omega_{0}z/v}.$$

For the sake of simplicity, we hereinafter do not explicitly indicate the dependence on the electron position vector **r**. The expansion coefficients $f_{\ell }^{n}$ in Eq. (2) can be written as

(4) $$f_{\ell }^{n}\left(r\right)=\alpha_{n+\ell }^{0}\;e^{i\tilde{\chi }}\;F_{\ell }^{n}$$

in terms of the matrix elements

(5) $$F_{\ell }^{n}=\left\langle n|\hat{S}\left(\sigma_{0}\right)\;e^{-i\lambda \left(a^{†}a+aa^{†}\right)/2}\hat{D}\left({\tilde{\beta }}_{0}\right)|n+\ell \right\rangle ,$$

where $\hat{S}\left(\sigma_{0}\right)=e^{\sigma_{0}\;aa/2-\sigma_{0}^{*}\;a^{†}a^{†}/2}$ and $\hat{D}\left({\tilde{\beta }}_{0}\right)=e^{{\tilde{\beta }}_{0}^{*}a^{†}-{\tilde{\beta }}_{0}a}$ are squeezing and displacement operators, respectively. The central operator $e^{-i\lambda \left(a^{†}a+aa^{†}\right)/2}$ is an exponential of the number operator, and therefore, it does not create or destroy excitations, so it just introduces a phase −(*n*+1/2)*λ* when applied on a Fock state |*n*⟩. This phase is often negligible when *θ*_0_ ∼ 0 (e.g., for a phase-matched interaction; see Section 2.1.2). Incidentally, an overall phase $\tilde{\chi }$ emerges in Eq. (4) as a result of the retarded elastic image interaction between the electron and the cavity [31], but such phase is irrelevant for this study. In Eq. (5), we introduce the squeezing and displacement coefficients

(6a) $$\sigma_{0}=arcsinh\left(\left|\nu \right|\right)\;e^{i\left(\arg\left\{\mu \right\}-\arg\left\{\nu \right\}\right)},$$

(6b) $$\begin{array}{ll} {\tilde{\beta }}_{0} & =\overset{z}{\underset{-\infty }{\int }}dz^{\prime }\left[e^{2i\theta_{0}\left(z^{\prime }\right)}\nu^{*}\left(z^{\prime }\right)\partial_{z^{\prime }}\beta_{0}^{*}\left(z^{\prime }\right)\right.\\ & \;\left.+e^{-2i\theta_{0}\left(z^{\prime }\right)}\mu^{*}\left(z^{\prime }\right)\partial_{z^{\prime }}\beta_{0}\left(z^{\prime }\right)\right], \end{array}$$

as well as *λ* = arg{*μ*}. These quantities are directly obtained from the solution of the Ricatti differential equation [32] ∂_*z*_ *R*/2 = k* − *kR* ^2^ with *R* = *ν*/*μ*,

(7a) $$\mu =\text{exp}\left\{2\overset{z}{\underset{-\infty }{\int }}dz^{\prime }k\left(z^{\prime }\right)R\left(z^{\prime }\right)\right\},$$

(7b) $$\nu =2\overset{z}{\underset{-\infty }{\int }}dz^{\prime }k^{*}\left(z^{\prime }\right)\mu \left(z^{\prime }\right),$$

and $k=i\sum_{i}\eta_{i}^{2}e^{4i\theta_{0}}$ . Interestingly, by differentiating Eq. (7), we readily verify that *μ* and *ν* satisfy the unitary condition of a generalized Bogoliubov transformation, |*μ*|^2^ − |*ν*|^2^ = 1 [33]. Also, when the ponderomotive force is neglected, the displacement factor ${\tilde{\beta }}_{0}$ becomes identical with the coupling factor found in Ref. [21]: $\beta_{0}=\left(e/\hbar \omega_{0}\right)\int_{-\infty }^{z}dz^{\prime }\;E_{0,z}\left(z^{\prime }\right)\;e^{-i\omega_{0}z^{\prime }/v}$ . We remark again that, given any distribution of the vector field ${\vec{E}}_{0}\left(r\right)$ , the function *k* can be computed and used to integrate the Ricatti differential equation and obtain the profile *R*. Then, Eq. (7) together with Eq. (6) provide a complete characterization of the electron–cavity interaction.

#### 2.1.2 Phase-matched interaction

Although the solution in Eq. (4) can be applied to any field profile, here we focus on a simple configuration that allows us to easily understand the problem, even though less symmetric scenarios could yield more efficient ponderomotive couplings. Specifically, we consider the electron to move along a path of length *L* while interacting with an electric field of the form ${\vec{E}}_{0}\left(r\right)={\vec{E}}_{0}\left(R\right)e^{iq_{z}z}$ , such that the phase velocity matches the electron velocity (i.e., *q*_*z*_ = *ω*_0_/*v*). In this scenario, which requires a specimen that possesses translational invariance along the e-beam direction, the ponderomotive coupling factor becomes independent of the longitudinal position *z*, so we have

$$k=i\underset{i}{\sum }\eta_{i}^{2}$$

**Figure 2:**
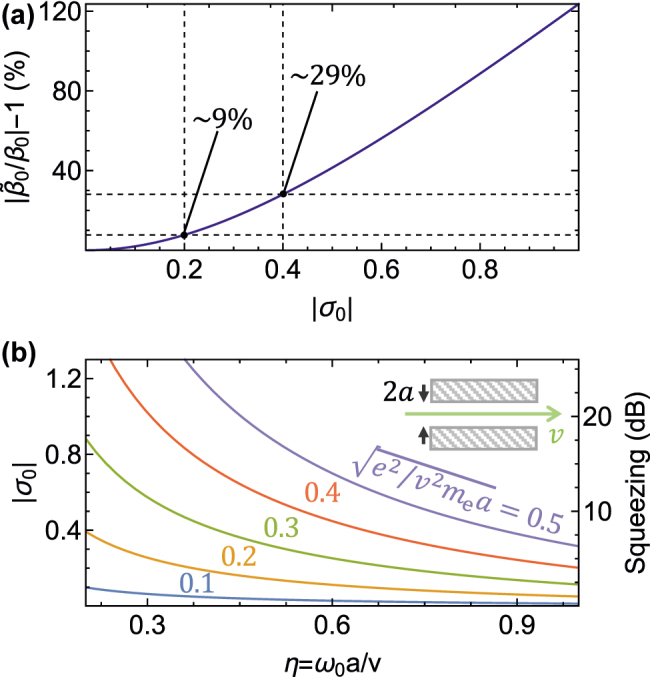
Linear and quadratic coupling in phase-matched interactions. (a) Deviation of the linear coupling coefficient $\left|{\tilde{\beta }}_{0}\right|$ from the value obtained by neglecting the ponderomotive force |*β*_0_| as a function of |*σ*_0_| for a phase-matched interaction with real *η*_*i*_ [see Eqs. (3) and (8a)]. (b) Squeezing parameter |*σ*_0_| (left vertical axis) and squeezing factor 20|*σ*_0_| dB (right vertical axis) as a function of *η* = *ω*_0_ *a*/*v* for different values of *v* ^2^ *a* under the electron–cavity configuration depicted in the inset, in which the e-beam moves with velocity *v* along the axis of a cylindrical hole of radius *a* and the cavity mode consists of a polariton made of a combination of azimuthal numbers *m* = ±1 and phase-matched axial wave vector *q*_*z*_ = *ω*_0_/*v*.

[see Eq. (3) for *η*_*i*_] by disregarding the small phase *θ*_0_. Then, the Ricatti equation admits an analytical solution that directly leads to the following compact forms of the coupling coefficients (see Methods, Section 4.2):

(8a) $${\tilde{\beta }}_{0}=\beta_{0}\;\left(1+2iL\underset{i}{\sum }|\eta_{i}{|}^{2}\right)+\beta_{0}^{*}\sigma_{0},$$

(8b) $$\sigma_{0}=2kL,$$

where $\beta_{0}=\left(eL/\hbar \omega_{0}\right)E_{0,z}\left(R\right)$ and *λ* = 0. Equation (8a) represents a generalization of the intrinsic PINEM coupling coefficient, now also incorporating the effect of the ponderomotive interaction. Interestingly, the presence of *σ*_0_ can play a significant role in the determination of the linear coupling and, in turn, in the computation of the related EELS probability given by $|{\tilde{\beta }}_{0}{|}^{2}$ , especially for long interaction lengths *L*. A quantitative estimate of such correction can be easily obtained for the special case in which all coefficients *η*_*i*_ are real, such that $|{\tilde{\beta }}_{0}/\beta_{0}|=\left|1+2i|\sigma_{0}|\right|$ . In Figure 2(a), we show that the deviation reaches ∼9 % for |*σ*_0_| ∼ 0.2. Besides the modification of the linear coupling, *σ*_0_ in Eq. (8a) reflects the magnitude of the effect produced by the squeezing operator [see Eq. (5)] on the electron–cavity dynamics.

A key point in this discussion refers to the normalization of the mode field ${\vec{E}}_{0}$ , which is determined by the integral $\int d^{3}r\;\epsilon \left(r\right)|{\vec{E}}_{0}\left(r\right){|}^{2}=2\pi \hbar \omega_{0}$ for dielectric cavities made of materials with real permittivity *ϵ*(**r**) [34]. For instance, for an electric field of real amplitude *E*_0_ uniformly distributed in vacuum over an area *A* and oriented perpendicularly with respect to the e-beam velocity, the normalization condition leads to $E_{0}=\sqrt{2\pi \hbar \omega_{0}/LA}$ , which in turn yields a squeezing factor |*σ*_0_| = *e* ^2^ *π*/*ω*_0_ *m*_e_ *vA*. Unfortunately, we immediately notice that the ponderomotive coupling coefficient is not proportional to the path length *L*, in contrast to the linear PINEM term ${\tilde{\beta }}_{0}$ arising when the field has a component along the *z* direction – a mechanism that has been argued to enable strong electron–cavity coupling and, from here, electron-mediated entanglement between different cavities [35]. Such different scaling suggests the use of modes lacking translational invariance along the electron trajectory and having a strong confinement in the tranverse directions (e.g., nanoscale gaps between two metallic nanoparticles [36] or a nanoparticle and a planar metallic surface [37]) as an approach to reach high squeezing values.

Nonetheless, the dependence of *σ*_0_ on the inverse of the electron velocity, the inverse mode frequency, and the field confinement (i.e., ∝ 1/*A*) should allow us to reach sizeable ponderomotive couplings by going to slow electrons interacting with infrared fields. Indeed, for a mode of energy *ℏω*_0_ = 15 meV distributed over an area *A* = 100 nm^2^, a 50 eV electron produces a squeezing parameter |*σ*_0_| ∼ 0.1.

As a potentially practical scenario, we consider the electron to be focused at the center of a circular hole (**R** = 0) of radius *a* drilled in a polaritonic material (see inset in Figure 2(b)) under the aforementioned phase-matching condition. In particular, for a mode consisting of a linear combination of two degenerate modes with azimuthal numbers *m* = ±1 and a relative phase *ψ*, the linear coupling vanishes (i.e., $E_{0z}=0$ ), while the ponderomotive coupling coefficient becomes *σ*_0_ = *i* e^i*ψ*^ *e* ^2^/2*m*_e_ *γv* ^2^ *aηI*(*η*) with *η* = *ω*_0_ *a*/*v* and *I*(*η*) standing for a normalization integral (see Methods, Section 4.2.1, for details). This dependence clearly favors small radii (i.e., strong field confinement), small electron velocities, and small resonance energies (see Figure 2(b)), in agreement with the scaling properties presented above.

### 2.2 Postinteraction quantum cavity state

#### 2.2.1 Cavity prepared in the ground state

We now discuss the photonic state after interaction with the electron for the cavity mode starting in the ground state (i.e., $\alpha_{n}^{0}=\delta_{n,0}$ ) and the e-beam being well focused around a transverse position **R**_0_, so that we can approximate |*ψ*_0_(**r**, *t*)|^2^ ≈ *δ*(**R** − **R**_0_)|*ϕ*_0_(*z* − *vt*)|^2^. In addition, because we are interested in the state of the system after the electron has abandoned the interaction region, we let the longitudinal coordinate *z* go to infinity in the calculation of all coupling parameters.

By starting from Eq. (2), we first obtain the full density matrix $\hat{\rho }\left(r,r^{\prime },t\right)=|\psi \left(r,t\right)〉〈\psi \left(r^{\prime },t\right)|$ , and then, by integrating over the electron coordinates, we find that the density matrix of the cavity mode reduces to $\int d^{3}r\;\hat{\rho }\left(r,r,t\right)=\sum_{nn^{\prime }}e^{i\left(n^{\prime }-n\right)\omega_{0}t}\rho_{nn^{\prime }}^{\text{ph}}|n〉〈n^{\prime }|$ , with matrix elements $\rho_{nn^{\prime }}^{\text{ph}}=M_{\omega_{0}\left(n^{\prime }-n\right)/v}\;e^{i\left(\theta_{n}-\theta_{n^{\prime }}\right)}F_{-n}^{n}F_{-n^{\prime }}^{n^{\prime }*}$ that involve the coherence factor $M_{\omega /v}=\int_{-\infty }^{\infty }dz\;|\phi_{0}\left(z\right){|}^{2}\;e^{i\omega z/v}$ . The latter, which only depends on the e-beam probability density and not on the phase of the wave function, determines the ability of the system to interfere with light synchronized with the electron pulse [38, 39]. We remark that this procedure renders the correct statistical properties of the cavity when no measurement is performed on the electron [40].

Interestingly, the coefficients $F_{-n}^{n}$ turn out to be the projections of the well-known two-photon coherent state defined in a seminal paper by Yuen [29], from which we find

(9a) $$\begin{array}{ll} \rho_{nn^{\prime }}^{\text{ph}} & =N^{2}\;M_{\omega_{0}\left(n^{\prime }-n\right)/v}\;e^{i\Delta_{nn^{\prime }}}{\left(n!n^{\prime }!\right)}^{-1/2}\\ & \;\times {\left(\tanh|\sigma_{0}|/2\right)}^{\left(n+n^{\prime }\right)/2}H_{n}\left(\zeta \right)H_{n^{\prime }}^{*}\left(\zeta \right), \end{array}$$

where *H*_*n*_ are Hermite polynomials and we have defined

(9b) $$\Delta_{nn^{\prime }}=\theta_{n}-\theta_{n^{\prime }}+\left(n^{\prime }-n\right)\;\arg\left\{\sigma_{0}\right\}/2,$$

(9c) $$\zeta =|{\tilde{\beta }}_{0}|e^{i\phi }/\sqrt{\sinh\left(2|\sigma_{0}|\right)},$$

(9d) $$\phi =\arg\left\{\sigma_{0}\right\}/2-\arg\left\{{\tilde{\beta }}_{0}\right\}-\lambda ,$$

as well as the normalization constant

(9e) $$N^{2}=e^{-|{\tilde{\beta }}_{0}{|}^{2}\left[1-\cos\left(2\phi \right)\tanh|\sigma_{0}|\right]}/\cosh|\sigma_{0}|.$$

**Figure 3:**
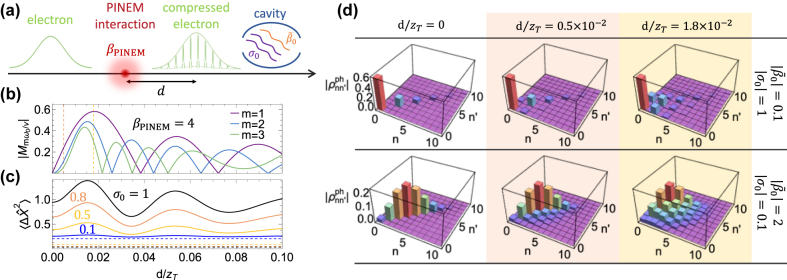
Postinteraction cavity-mode population. (a) Sketch of an electron wave packet undergoing a classical PINEM interaction of strength *β*_PINEM_, and subsequently propagating in free-space for a distance *d*, where it develops a sequence of probability-density pulses and interacts with a cavity with coupling coefficients *σ*_0_ and ${\tilde{\beta }}_{0}$ . (b) Coherence factor $\left|M_{m\omega_{0}/v}\right|$ , determining the amount of coherence left in the two-photon coherent state [see Eq. (9a)] for the scenario depicted in panel (a) with *β*_PINEM_ = 4. We plot the result as a function of the normalized propagation distance *d*/*z*_T_ for several harmonics *m*. (c) Position variance $\left\langle \Delta {\hat{X}}^{2}\right\rangle$ for a PINEM-compressed electron [solid curves; see panel (a)] and for a perfectly coherent electron (dashed curves). We consider different real values of *σ*_0_ with $\tilde{\beta }=0$ , *θ*_*n*_ = 0, and *λ* = 0. (d) Elements $\rho_{nn^{\prime }}^{\text{ph}}$ of the density matrix associated with the cavity state after interaction with a compressed electron [see panel (a)]. The interaction region is taken to be placed at the propagation distances highlighted by the color-coordinated vertical dashed lines in panel (b) assuming pairs of coupling parameters $|{\tilde{\beta }}_{0}|=0.1$ and |*σ*_0_| = 1 (top row); or $|{\tilde{\beta }}_{0}|=2$ , |*σ*_0_| = 0.1 (bottom row). All plots in panel (d) are calculated for *ϕ* = 0.

Equation (9a) tells us that different postinteraction cavity states can be selected by properly tuning the factors ${\tilde{\beta }}_{0}$ and *k*. For instance, in a geometry for which ${\vec{E}}_{0}\cdot v∼0$ , as in the case analyzed in the previous section, where an electron traverses a circular hole, the coupling coefficient ${\tilde{\beta }}_{0}$ vanishes and the state assumes the form of a squeezed vacuum containing only an even number of photons in the diagonal of the density matrix. This conclusion can be easily verified by evaluating the Hermite polynomials at zero argument or, alternatively, directly from Eq. (4). In contrast, the off-diagonal elements bear a more intricate dependence on the incoming electron wave function given by the coherence factor $M_{\omega_{0}m/v}$ , which determines the possibility of creating squeezed states with the correct coherences. For example, an electron that has undergone a PINEM interaction with an optical field of photon energy *ℏω*_0_ and coupling strength |*β*_PINEM_|, followed by free propagation along a macroscopic distance *d* (see Figure 3(a)), has an associated probability density consisting of a train of pulses described by the coherence factor

$$\begin{array}{ll} M_{\omega_{0}m/v} & =e^{2\pi im^{2}d/z_{\text{T}}}\sum_{\ell }J_{\ell }\left(2|\beta_{\text{PINEM}}|\right)\\ & \;\times J_{\ell +m}\left(2|\beta_{\text{PINEM}}|\right)e^{4\pi im\ell d/z_{\text{T}}}\\ & =i^{m}J_{m}\left[4|\beta_{\text{PINEM}}\text{sin}(2\pi md/z_{\text{T}})|\right]\\ & \;\times {\left[\text{sgn}\left\{\text{sin}(2\pi md/z_{\text{T}})\right\}\right]}^{m} \end{array}$$

where the phase of the PINEM interaction has been set to zero, *z*_T_ = 4*πm*_e_ *v* ^3^ *γ* ^3^/*ℏω*_0_ is the so-called Talbot distance [38, 41], and *J*_*ℓ*_ is the Bessel function of order *ℓ*. We show the resulting coherence factor in Figure 3(b). The corresponding state left in the cavity after interacting with the electron (top row of Figure 3(c)) comprises off-diagonal elements that become visible only when $\left|M_{\omega_{0}m/v}\right|$ takes values around its maximum along the propagation distance ( $|M_{\omega_{0}m/v}|∼0.58$ for *m* = 1). Despite this limitation, values of $|M_{\omega_{0}m/v}|∼1$ have been shown to be realizable by means of a sequence of several cycles of PINEM interaction and free-space propagation [42]. From the expression of *σ*_0_ related to such interaction, and by using the state in Eq. (9a) with full electron coherence $\left(M_{\omega_{0}m/v}=1\right)$ as well as by setting ${\tilde{\beta }}_{0}=0$ , *λ* = 0 and *θ*_*n*_ = 0, we compute the variance $\left\langle \Delta {\hat{X}}^{2}\right\rangle =\left\langle {\hat{X}}^{2}\right\rangle -{\left\langle \hat{X}\right\rangle }^{2}$ with $\hat{X}=\left(a+a^{†}\right)/2$ – a quantity of utmost importance, which represents the fluctuations in the position of the harmonic oscillator (HO) for the cavity mode. By choosing *ψ* = −*π*/2 to make *σ*_0_ real, we obtain $\left\langle \Delta {\hat{X}}^{2}\right\rangle =e^{-2|\sigma_{0}|}/4$ . In Figure 2(b), we present the degree of squeezing in decibel (vertical right axis) through the relation $-10\log\left(4\left\langle \Delta {\hat{X}}^{2}\right\rangle \right)=20|\sigma_{0}|$ . Interestingly, the squeezing approaches values as high as ∼10 dB for a 5 eV electron, *a* ∼ 3 nm, and a resonance energy *ℏω*_0_ ∼ 60 meV; this squeezing value represents a threshold of particular importance to grant us access into fault-tolerant quantum algorithms by means of GKB codes [43]. High values of |*σ*_0_| can also be obtained for larger electron energies (e.g., ≳70 eV for *a* ≳ 1 nm) by targeting mid-infrared excitations (e.g., phonon polaritons in hexagonal boron nitride, which exhibit high quality factors even when structured with a lateral size of a few nanometers [44]).

We also consider the effect of having a partially coherent electron. In particular, by compressing the electron through free-space propagation following a single PINEM interaction (setting again ${\tilde{\beta }}_{0}=0$ , *λ* = 0, and *θ*_*n*_ = 0), we directly compute $\left\langle \Delta {\hat{X}}^{2}\right\rangle$ by using the matrix elements in Eq. (9a) and find the results presented in Figure 3(c), which clearly show an increase in the position uncertainty with respect to the values obtained for perfectly coherent electrons (e.g., when treated as point particles). In addition, it is interesting to note that $\left\langle \Delta {\hat{X}}^{2}\right\rangle$ increases with |*σ*_0_| as a consequence of the vanishing coherence factor $M_{\omega_{0}m/v}$ at the harmonics connected to the populated Fock states |*n*⟩.

In the opposite extreme (*k* → 0), from Eqs. (6a) and (7), we obtain |*σ*_0_| → 0, which leads to a Poissonian distribution in $P_{n}=\rho_{nn}^{\text{ph}}$ by applying the asymptotic form of *H*_*n*_(*ζ*) or, again, by simply taking the same limit in Eq. (4). This behavior is particularly evident in the bottom row of Figure 3(c), where we plot the mode population obtained upon direct evaluation of Eq. (9a) for different propagation distances from the first PINEM interaction.

For parameters ${\tilde{\beta }}_{0}$ and *σ*_0_ lying in between the two extremes under consideration, the cavity state varies in a continuous fashion, spanning values of the zero-delay second-order correlation function g(2)(0)=∑nn(n−1)Pn∑nnPn2 ranging from a super-Poissonian statistics with *g* ^(2)^(0) ∼ 3 + sinh^−2^(|*σ*_0_|) to a Poissonian statistics with *g* ^(2)^(0) ∼ 1 when the phase shift is *ϕ* = *π*/2 [see Eq. (9d)], as well as to a sub-Poissonian statistics for *ϕ* = 0. This is a consequence of the decreasing variance of the distribution of photon numbers, $\Delta n^{2}=2\;\sinh^{2}\left(\left|\sigma_{0}\right|\right)\cosh^{2}\left(\left|\sigma_{0}\right|\right)+|{\tilde{\beta }}_{0}{|}^{2}\left[cosh\left(4|\sigma_{0}|\right)-\cos\left(2\phi \right)sinh\left(4|\sigma_{0}|\right)\right]$ . We remark that the exponential dependence of the variance on the squeezing parameter renders the postinteraction cavity state extremely sensitive to any change in the phase *ϕ*, even for small |*σ*_0_|, provided the interaction is assisted by a high coupling with the electric field component along the e-beam direction.

#### 2.2.2 Cavity under laser illumination

Although the optical cavity mode may in principle be chosen such that it constrains the phase *ϕ* [Eq. (9d)], in practical configurations this parameter would be hard to change in a single experimental setup. A more feasible route to gain control over the phase *ϕ* consists in aligning the e-beam such that $E_{0,z}=0$ while the cavity is irradiated by a laser. Intuitively, the postinteraction cavity state should take a form that resemblances the one in Eq. (9a), but with ${\tilde{\beta }}_{0}$ changed to a factor *α*_L_ proportional to the incident laser amplitude and carrying its phase. More precisely, the probability to leave *n* photons in the cavity is

(10) $$P_{n}=\sum_{\ell ,\ell^{\prime }}M_{\omega_{0}\left(\ell^{\prime }-\ell \right)/v}F_{\ell }^{n}F_{\ell^{\prime }}^{n*}\times \langle n+\ell |\hat{D}\left(\alpha_{\text{L}}\right)|0\rangle \langle 0|{\hat{D}}^{†}\left(\alpha_{\text{L}}\right)|n+\ell^{\prime }\rangle .$$

In contrast to the scenario with no external pumping, the probabilities *P*_*n*_ in Eq. (10) are strongly dependent on the electron coherence factor. Specifically, Eq. (10) reduces to Eq. (9a) only if the electron bears full coherence with respect to the laser [i.e., for $M_{\omega_{0}\left(\ell^{\prime }-\ell \right)/v}=1$ ].

### 2.3 Electron spectrum after interaction with an excited cavity

We now study the electron spectrum resulting from the interaction with a cavity that is exposed to a synchronized light source. We model this scenario by assuming different initial cavity states (i.e., Fock-state amplitudes $\alpha_{n}^{0}$ ) and an electron whose temporal envelope varies negligibly during one optical period (e.g., for a ∼100 fs electron pulse and a mode energy *ℏω*_0_ ∼ 1 eV, or equivalently, a mode period 2*π*/*ω*_0_ ≈ 4.1 fs). Under these conditions, once the electron has abandoned the interaction region, its state is approximately given by a discrete superposition of plane waves of energies *E*_0_ + *ℏω*_0_ *ℓ*/*v* labeled by the net number of exchanged photons *ℓ*, with associated intensities

(11) $$P_{\ell }=\overset{\infty }{\underset{n=0}{\sum }}|f_{\ell }^{n}{|}^{2}.$$

The resulting electron energy-loss distribution is $\Gamma \left(\omega \right)=\sum_{\ell =-\infty }^{\infty }P_{\ell }\;\delta \left(\omega +\ell \omega_{0}\right)$ , as obtained directly from Eq. (2) by setting *ϕ*_0_(**r** − **v** *t*) = (2*π*)^−3/2^ and recalling that, in the nonrecoil approximation, the energy is only dependent on the momentum component parallel to the electron trajectory.

We first consider a quasi-monochromatic laser pulse with central frequency *ω*_0_ irradiating the cavity and inducing an initial coherent state with coefficients $\alpha_{n}^{0}=e^{-|\alpha_{\text{L}}{|}^{2}/2}\alpha_{\text{L}}^{n}/\sqrt{n!}$ in the targeted cavity mode. Neglecting any nonlinear response of the cavity materials, we have that *α*_L_ is proportional to the electric field of the incident laser pulse. In practice [16, 45], the light electric field can be as high as $∼10^{8}$ V/m and produce an average population reaching $\bar{n}=\sum_{n}n|\alpha_{n}^{0}{|}^{2}∼300$ excitations for a mode of energy *ℏω*_0_ = 1 eV and uniform spatial distribution over a volume of 10^−3^ μm^3^. In this limit, the Poissonian distribution $|\alpha_{n}^{0}{|}^{2}$ approaches a Gaussian distribution with equal average and standard deviation corresponding to |*α*_L_|^2^, which we further approximate as a Kronecker delta peaked at the integer nearest to $\bar{n}$ . Interestingly, also in the $\bar{n}\gg 1$ limit, and assuming small linear and quadratic coupling coefficients $\left|{\tilde{\beta }}_{0}\right|$ and |*σ*_0_| such that they do not produce large interaction probabilities for $\ell ∼\bar{n}$ , we can write the $F_{\ell }^{n}$ coefficients in Eq. (4) as (see Methods, Section 4.3)

(12) $$\begin{array}{ll} F_{\ell }^{n} & \approx e^{i\ell \arg\left\{-{\tilde{\beta }}_{0}\right\}-i\lambda \left(n+1/2\right)}\sum_{n=-\infty }^{\infty }e^{-2in\phi }\\ & \;\times J_{-n}\left[\left(n+\ell \right)|\sigma_{0}|\right]J_{2n+\ell }\left[2\sqrt{n+\ell }\;|\;{\tilde{\beta }}_{0}|\right]. \end{array}$$

Reassuringly, Eq. (12) reproduces the result obtained for an electron interacting with two mutually coherent fields of frequencies *ω*_0_ and 2*ω*_0_ [17, 46]. Then, plugging this expression into Eq. (11), we obtain the electron probabilities

(13) $$P_{\ell }^{\text{coh}}=\sum_{n,n^{\prime }=-\infty }^{\infty }e^{2i\left(n^{\prime }-n\right)\phi }\;I_{nn^{\prime }\ell }\left(1\right),$$

where $I_{nn^{\prime }\ell }\left(x\right)=J_{-n}\left(x|\sigma |\right)\;J_{2n+\ell }\left(2\sqrt{x}|\tilde{\beta }|\right)\;J_{-n^{\prime }}\left(x|\sigma |\right)J_{2n^{\prime }+\ell }\left(2\sqrt{x}|\tilde{\beta }|\right)$ , and we introduce the parameters $\left|\tilde{\beta }|=\sqrt{\bar{n}}|{\tilde{\beta }}_{0}\right|$ and $\left|\sigma |=\bar{n}|\sigma_{0}\right|$ connecting the one-mode classical coupling parameters to their quantum counterparts $\left|{\tilde{\beta }}_{0}\right|$ and |*σ*_0_|.

**Figure 4:**
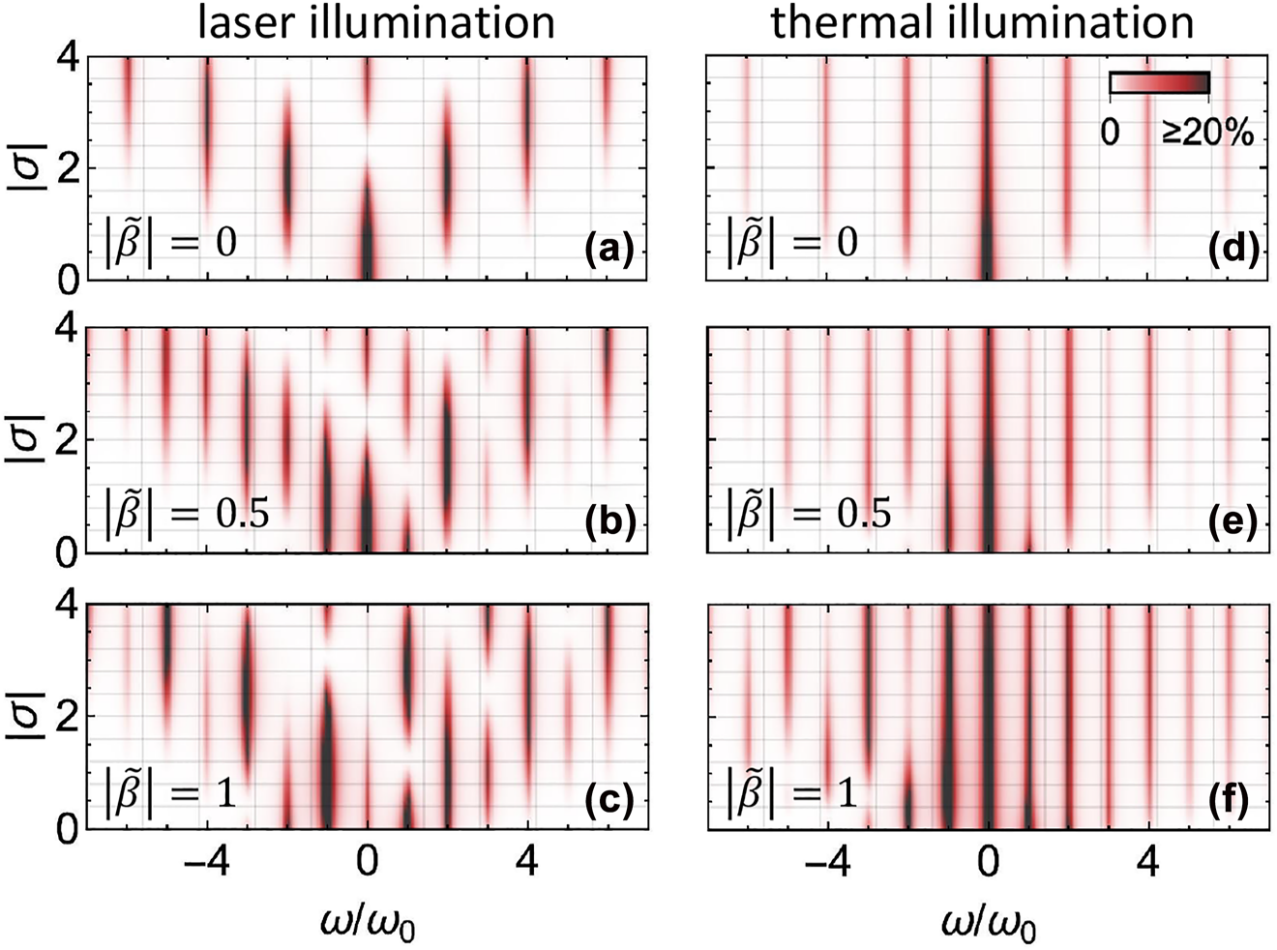
Electron energy-loss spectrum. Electron distribution as a function of the normalized energy loss *ω*/*ω*_0_ and the ponderomotive coupling |*σ*| for $|\tilde{\beta }|=0$ (a, d), $|\tilde{\beta }|=0.5$ (b, e), and $|\tilde{\beta }|=1$ (c, f). We take the cavity to be initially prepared in either a coherent state (a–c) or a thermal state (d–f), as described by Eqs. (13) or (14), respectively. The conditions $\bar{n}\gg 1$ and *ϕ* = *π*/2 are assumed in all panels, and a Lorentzian broadening in *ω* (FWHM = 0.12*ω*_0_) is introduced for clarity.

In Figure 4(a–c), we show electron spectra calculated from Eq. (13) as a function of *σ* and the normalized energy loss *ω*/*ω*_0_ for selected values of $\tilde{\beta }$ . For vanishing linear coupling $\left(\tilde{\beta }=0\right)$ , we observe the emergence of several energy sidebands separated by multiples of 2*ℏω*_0_, as expected from the quadratic interaction with the field. Like in PINEM, the sidebands are symmetrically distributed around the zero-loss peak. In addition, as |*σ*| increases, we observe oscillations in the intensities, also similar to those in PINEM [16], and equally resulting from the interaction between the electron and the coherently excited mode. Once the linear interaction is turned on $\left(\tilde{\beta }>0\right)$ , the interference with quadratic-coupling channels manifests in an asymmetric peak distribution, which is already discernible for small |*σ*| values and can be controlled by tuning the relative phase *ϕ*. This effect was observed in previous theoretical works by numerically integrating the Schrödinger equation for a Hamiltonian incorporating the interaction of a free electron with a classical light field [26].

Similarly, a highly populated mode can be also obtained via cavity heating at temperatures such that *k*_B_ *T*/*ℏω*_0_ ≫ 1. In this regime, the cavity is prepared in a pure mixture with a classical Boltzmann probability distribution, which is well approximated by the expression $p_{n_{0}}\approx e^{-n_{0}/\bar{n}}/\bar{n}$ with $\bar{n}\approx k_{B}T/\hbar \omega_{0}$ . We calculate the probability of exchanging an energy *ℓℏω*_0_ by setting $\alpha_{n+\ell }^{0}=\delta_{n+\ell ,n_{0}}$ in Eq. (12) and then summing over all the possible values of the initial number of photons *n*_0_, weighted by their respective populations $p_{n_{0}}$ . Since $\bar{n}\gg 1$ , we approximate this sum to an integral taken over the large extension of the distribution (see Ref. [21]). Thus, for a thermally excited cavity mode interacting with the electron, we obtain

(14) $$P_{\ell }^{\text{th}}=\overset{\infty }{\underset{n,n^{\prime }=-\infty }{\sum }}e^{i2\left(n^{\prime }-n\right)\phi }\;\overset{\infty }{\underset{0}{\int }}dx\;e^{-x}\;I_{nn^{\prime }\ell }\left(x\right).$$

To compare this expression with the results obtained under coherent laser illumination, we evaluate Eq. (14) and plot Figure 4(d–f). As one could infer directly from the form of Eq. (14), the effect of the thermal population is to smear the electron spectra obtained under coherent illumination. As a consequence, the intensity oscillations observed in Figure 4(a–c) with increasing |*σ*| are absent in Figure 4(d–f) and just replaced by a monotonic decrease with |*σ*|.

## 3 Concluding remarks

In brief, the inclusion of *A* ^2^ terms in the interaction between e-beams and optical cavity modes causes a departure from the role of electrons as semiclassical excitation sources. In the absence of such terms, free electrons can only create coherent states by interaction with bosonic optical modes [20], unless a postselection of the electron state is performed [6]. In contrast, the presence of a ponderomotive coupling component embodied in the *A* ^2^ terms gives rise to a new set of cavity states that include vacuum-, phase-, and amplitude-squeezed states, directly created by interaction with the electron without the need of any postselection. We also demonstrate that a squeezing of ∼10 dB, which represents the current fundamental limit for the implementation of quantum algorithms by means of nonclassical states of light, can be produced in realistic designs of nanostructured polaritonic materials.

In addition, our work shows that the ponderomotive terms allow us to estimate the linear coupling strength ${\tilde{\beta }}_{0}$ , especially when this parameter reaches large values as a consequence of strong electron–cavity coupling, a condition that has been achieved in recent experiments by phase-matching the electron–cavity mode interaction [47–49] and is being theoretically investigated to be implemented in integrated photonic designs [50].

We understand that the present work paves the way toward the generation of nonclassical states of light with nanometric precision, even in dark modes that do not couple to light, thus revealing a new tuning knob at the intersection of quantum information and e-beam technologies.

## 4 Methods

### 4.1 Theoretical description of the quantum electron–cavity interaction in the nonrecoil approximation

#### 4.1.1 The effective Hamiltonian

We start by considering a fast electron interacting with a classical electromagnetic field described in the temporal gauge (i.e., with zero scalar potential) in the presence of a material structure. In our analysis, the initial electron wave function is assumed to be in a superposition of momenta concentrated around a value *ℏ* **k**_0_, which corresponds to a relativistic energy *E*_0_ and velocity **v** = *ℏ* **k**_0_ *c* ^2^/*E*_0_. Under these conditions, and for optical excitations in the infrared-visible range, the system dynamics can be modeled by means of the effective Schrödinger equation $i\hbar \partial_{t}\psi \left(r,t\right)=H\left(t\right)\psi \left(r,t\right)$ with the minimal-coupling Hamiltonian [51]

$$H\left(t\right)=E_{0}-\hbar v\cdot \left(i\nabla +k_{0}\right)+\left(ev/c\right)\cdot A\left(r,t\right)+\underset{i=x,y,z}{\sum }g_{i}A_{i}^{2}\left(r,t\right),$$

where **A**(**r**, *t*) is the time-dependent vector potential associated with the external illumination in the presence of the cavity. The coefficient vector **g** = (*e* ^2^/2*m*_e_ *c* ^2^ *γ*)(1, 1, *γ* ^−2^) in the ponderomotive term approximately incorporates relativistic corrections through the Lorentz factor $\gamma =1/\sqrt{1-v^{2}/c^{2}}$ . The electron motion is then described by the scalar wave function *ψ*(**r**, *t*).

In this work, we are interested in a quantum description of the cavity and, thus, adopt a quantum-optics approach [34] with the vector potential treated as an operator: $\hat{A}\left(r\right)=\sum_{j}\left(-ic/\omega_{j}\right)\left[{\vec{E}}_{j}\left(r\right)a_{j}-{\vec{E}}_{j}^{*}\left(r\right)a_{j}^{†}\right]$ , where *j* runs over cavity modes, ${\vec{E}}_{j}\left(r\right)$ are the corresponding normalized electric field distributions, and we introduce bosonic annihilation and creation operators *a*_*j*_ and $a_{j}^{†}$ . By further assuming the sample to undergo negligible inelastic losses, and focusing on the interaction with a spectrally dominant and well-defined mode (labeled by *j* = 0, and for simplicity we dismiss the *j* label in the ladder operators), the electron–cavity system can be described by the modified Schrödinger equation $i\hbar \partial_{t}|\psi \left(r,t\right)〉=\left({\hat{H}}_{0}+{\hat{H}}_{\text{int}}\right)|\psi \left(r,t\right)〉$ with

(15a) $${\hat{H}}_{0}=\hbar \omega_{0}a^{†}a+E_{0}-\hbar v\cdot \left(i\nabla +k_{0}\right),$$

(15b) $$\begin{array}{ll} {\hat{H}}_{\text{int}} & =\left(iev/\omega_{0}\right)\cdot \left[{\vec{E}}_{0}^{*}\left(r\right)a^{†}-{\vec{E}}_{0}\left(r\right)a\right]\\ & \;-\underset{i}{\sum }\left(c^{2}g_{i}/\omega_{0}^{2}\right){\left[E_{0,i}\left(r\right)a-E_{0,i}^{*}\left(r\right)a^{†}\right]}^{2}, \end{array}$$

where we have added the noninteracting Hamiltonian of the cavity mode *ℏω*_0_ *a* ^†^ *a* and included the photonic degrees of freedom in the combined state $|\psi \left(r,t\right)〉=\sum_{n=0}^{\infty }\psi_{n}\left(r,t\right)e^{-i\omega_{0}nt}|n〉$ .

#### 4.1.2 Exact analytical solution

The interaction Hamiltonian in Eq. (15b) presents a quadratic term in the mode ladder operators; therefore, generating a dynamics that substantially differs from the one addressed in previous works [16, 35] where only the linear coupling with the electromagnetic field was taken into account. Despite this difference, by following similar steps as those used in Ref. [21], we find an analytical solution for the combined wave function.

We start by inserting the ansatz $\psi_{n}\left(r,t\right)=\psi_{0}\left(r,t\right)\sum_{\ell =-\infty }^{\infty }f_{\ell }^{n}\left(r\right)e^{i\theta_{n}}e^{i\omega_{0}\ell \left(z/v-t\right)}$ into the Schrödinger equation, where $\psi_{0}\left(r,t\right)=e^{-iE_{0}t/\hbar +ik_{o}\cdot r}\phi_{0}\left(r-vt\right)$ is the initial electron wave function, $\theta_{n}=-\sum_{i}\int_{-\infty }^{z}dz^{\prime }|\eta_{i}{|}^{2}\left(2n+1\right)$ , $\eta_{i}=\sqrt{\hbar /2m_{e}v\gamma }\;\mu_{i}$ , and $\mu_{i}=\left(e/\hbar \omega_{0}\right)E_{0,i}e^{-i\omega_{0}z/v}$ . This leads to the set of one-dimensional differential equations

(16) $$\begin{array}{ll} \partial_{z}f_{\ell }^{n} & =p^{*}\sqrt{n}f_{\ell +1}^{n-1}-k^{*}\sqrt{n\left(n-1\right)}f_{\ell +2}^{n-2}\\ & \;-p\sqrt{n+1}f_{\ell -1}^{n+1}+k\sqrt{\left(n+1\right)\left(n+2\right)}f_{\ell -2}^{n+2}, \end{array}$$

with $p=\mu_{z}e^{2i\theta_{0}}$ and $k=i\sum_{i}\eta_{i}^{2}e^{4i\theta_{0}}$ .

Importantly, Eq. (16) conserves the total number of excitations, and thus, the set of coefficients defined by *ℓ* + *n* = *s* for each choice of an integer *s* evolves separately. This property allows us to map our problem onto a quantum harmonic oscillator under nonlinear coupling. Indeed, given the Hamiltonian ${\hat{H}}_{\text{HO}}={\hat{H}}_{\text{HO}}^{0}+g_{1}\left(t\right)\;a+g_{1}^{*}\left(t\right)\;a^{†}+g_{2}\left(t\right)\;a^{2}+g_{2}^{*}\left(t\right)\;a^{†2}$ , with ${\hat{H}}_{\text{HO}}^{0}=\hbar \omega_{0}a^{†}a$ , and plugging the general state $|\psi \left(t\right)〉=\sum_{n}c_{n}\left(t\right)e^{-in\omega_{0}t}|n〉$ into the associated Schrödinger equation, we obtain

(17) $$\begin{array}{ll} i\hbar \partial_{t}c_{n} & =\sqrt{n}c_{n-1}g_{1}^{*}e^{i\omega_{0}t}+\sqrt{n\left(n-1\right)}c_{n-2}g_{2}^{*}e^{2i\omega_{0}t}\\ & \;+\sqrt{n+1}c_{n+1}g_{1}e^{-i\omega_{0}t}+\sqrt{\left(n+2\right)\left(n+1\right)}c_{n+2}g_{2}e^{-2i\omega_{0}t}, \end{array}$$

which connects to Eq. (16) by means of the transformation $c_{n}\to f_{s-n}^{n}$ , *t* → *z*, $ig_{1}e^{-i\omega_{0}t}/\hbar \to p$ , and $-ig_{2}e^{-2i\omega_{0}t}/\hbar \to k$ . The coefficients *c*_*n*_(*t*) can also be expressed in terms of the scattering operator $\hat{S}\left(t,-\infty \right)$ as $c_{n}\left(t\right)=\sum_{m=0}^{\infty }\left\langle n|\hat{S}\left(t,-\infty \right)|m\right\rangle c_{m}\left(-\infty \right)$ , assuming an initial state of the harmonic oscillator written here in the interaction picture [52] as |*ψ*(−∞)⟩_I_ = ∑_*m*_ *c*_*m*_(−∞)|*m*⟩. Consequently, we proceed by evaluating the matrix elements of the scattering operator.

To find the scattering operator, we first notice that an analytical solution to the equation $i\hbar \partial_{t}\;\hat{U}\left(t,t_{0}\right)={\hat{H}}_{\text{HO}}\;\hat{U}\left(t,t_{0}\right)$ was found by Yuen [29] and is given by [33]

$$\begin{array}{ll} \hat{U}\left(t,t_{0}\right) & =e^{i\tilde{\chi }}\;\hat{S}\left(\sigma_{0}e^{2i\omega_{0}t}\right)e^{-i\lambda \left(a^{†}a+aa^{†}\right)/2}\\ & \;\times e^{-i{\hat{H}}_{\text{HO}}^{0}\left(t-t_{0}\right)/\hbar }\hat{D}\left({\tilde{\beta }}_{0}e^{i\omega_{0}t_{0}}\right), \end{array}$$

where we have defined the operators $\hat{S}\left(z\right)=e^{z\;aa/2-z^{*}a^{†}a^{†}/2}$ and $\hat{D}\left(z\right)=e^{z^{*}a^{†}-za}$ , the linear coupling ${\tilde{\beta }}_{0}=\left(i/\hbar \right)\int_{t_{0}}^{t}dt^{\prime }\left(g_{1}\left(t^{\prime }\right)e^{i\omega_{0}t^{\prime }}-g_{1}^{*}\left(t^{\prime }\right)e^{i\omega_{0}t^{\prime }}\nu^{*}\right)$ , and the quadratic coupling *σ*_0_ = arcsinh(|*ν*|)e^i(arg{*μ*}−arg{*ν*})^. The coefficients *μ* = e^i*ϕ*^ and $\nu =\left(2i/\hbar \right)\int_{t_{0}}^{t}dt^{\prime }g_{2}^{*}\left(t^{\prime }\right)e^{2i\omega_{0}t^{\prime }}\mu \left(t^{\prime }\right)$ , with $\phi =\left(-2/\hbar \right)\int_{t_{0}}^{t}dt^{\prime }\;g_{2}\left(t^{\prime }\right)e^{-2i\omega_{0}t^{\prime }}R\left(t^{\prime }\right)$ , satisfy the relation |*μ*|^2^ − |*ν*|^2^ = 1 at every time, while the function *R* = *ν*/*μ* obeys the Ricatti equation [32] ∂_*t*_ *R*/2 = k* − *k R* ^2^ solved with the initial condition *R*(*t*_0_) = 0. We have also defined the phase *λ* = arg{*μ*} and introduced the phase $\tilde{\chi }=\left(i/2\right)\int_{t_{0}}^{t}dt^{\prime }\left({\tilde{\beta }}_{0}^{*}\partial_{t^{\prime }}{\tilde{\beta }}_{0}-{\tilde{\beta }}_{0}\partial_{t^{\prime }}{\tilde{\beta }}_{0}^{*}\right)$ , which is a generalization of the Berry phase found in a forced quantum harmonic oscillator [31, 53].

At this point, we invoke the relation $\hat{S}\left(t,t_{0}\right)=e^{i{\hat{H}}_{\text{HO}}^{0}t/\hbar }\;\hat{U}\left(t,t_{0}\right)\;e^{-i{\hat{H}}_{\text{HO}}^{0}t_{0}/\hbar }$ , as well as $e^{i{\hat{H}}_{\text{HO}}^{0}t_{0}/\hbar }\hat{D}\left({\tilde{\beta }}_{0}e^{i\omega_{0}t_{0}}\right)e^{-i{\hat{H}}_{\text{HO}}^{0}t_{0}/\hbar }=\hat{D}\left({\tilde{\beta }}_{0}\right)$ and $e^{i{\hat{H}}_{\text{HO}}^{0}t/\hbar }\hat{S}\left(\sigma_{0}e^{2i\omega_{0}t}\right)e^{-i{\hat{H}}_{\text{HO}}^{0}t/\hbar }=\hat{S}\left(\sigma_{0}\right)$ , which can be easily verified by using ordering theorems for the displacement operator $\hat{D}\left({\tilde{\beta }}_{0}\right)=e^{-|{\tilde{\beta }}_{0}{|}^{2}/2}e^{{\tilde{\beta }}_{0}^{*}a^{†}}e^{-{\tilde{\beta }}_{0}a}$ and the squeezing operator $\hat{S}\left(\sigma_{0}\right)=\exp\left[-\text{tanh}\left(\left|\sigma_{0}\right|\right)e^{-i\arg\left\{\sigma_{0}\right\}}a^{†}a^{†}/2\right]$ $\exp\left\{-\log\left[\text{cosh}\left(\left|\sigma_{0}\right|\right)\right](a^{†}a+aa^{†})/2\right\}\exp\left[\text{tanh}\left(\left|\sigma_{0}\right|\right){\text{e}}^{i\arg\left\{\sigma_{0}\right\}}aa/2\right]$ , respectively [54]. By employing these expressions, we find

(18) $$\hat{S}\left(t,t_{0}\right)=e^{i\tilde{\chi }}\;e^{\sigma_{0}\;aa/2-\sigma_{0}^{*}a^{†}a^{†}/2}\;e^{-i\lambda \left(a^{†}a+aa^{†}\right)/2}\;e^{{\tilde{\beta }}_{0}^{*}a^{†}-{\tilde{\beta }}_{0}a}.$$

Finally, once the aforementioned transformation is applied to Eq. (18) and the *t*_0_ → −∞ limit is taken, the coefficients $f_{\ell }^{n}$ in Eq. (4) of the main text are found by imposing the boundary condition $\psi_{n}\left(z\to -\infty ,t\right)=\psi_{0}\left(r,t\right)\sum_{n=0}\alpha_{n}^{0}e^{-in\omega_{0}t}|n〉$ , which is equivalent to $f_{\ell }^{n}\left(z\to -\infty \right)=\delta_{\ell ,0}\alpha_{n}^{0}$ .

### 4.2 Phase-matched interaction

For an electron interacting with a structure that is translationally invariant along the e-beam propagation direction *z*, the electric field of the mode assumes the general functional form $E_{0,i}\left(r\right)=E_{0,i}\left(R\right)e^{iq_{z}z}$ , with **R** = (*x*, *y*). We consider a guided polariton or a traveling wave of subluminal phase velocity in a high-refractive-index dielectric, such that the condition *ω*_0_/*v* = *q*_*z*_ is met by tuning the electron velocity. This condition has been previously explored as a way to enhance the electron-mode coupling amplitude [6], which in the absence of ponderomotive interactions renders $\tilde{\beta }\propto \sqrt{L}$ , where *L* is the interaction length [35]. In this scenario, we find the function $k=i\sum_{i}\eta_{i}^{2}$ with $\eta_{i}^{2}=\left(g_{i}e^{2}/\hbar \omega_{0}^{2}m_{e}v\gamma \right)E_{0,i}^{2}\left(R\right)$ , which is independent of *z* if we assume *θ*_0_ ∼ 0. This leads to the analytical solution *R* = e^−i arg{*k*}^tanh[2|*k*|(*z* + *L*/2)] for the Ricatti equation in Methods, Section 4.1.2. By plugging *R* into the coefficients *μ* and *ν*, and then integrating over *z* along the sample extension *L*, we obtain *μ* = cosh[2|*k*|*L*] and *ν* = e^−i arg{*k*}^sinh[2|*k*|*L*], from which the squeezing parameter reduces to Eq. (8b) in the main text (i.e., linear in $\eta_{i}^{2}$ ). To estimate ${\tilde{\beta }}_{0}$ up to the same order in $\eta_{i}^{2}$ , we cannot disregard the phase *θ*_0_ in the calculation. Then, we obtain Eq. (8a), which approaches $\beta_{0}=\left(eL/\hbar \omega_{0}\right)E_{0,z}\left(R\right)$ for vanishing $\eta_{i}^{2}$ .

#### 4.2.1 Hole in a metallic slab

In order to estimate the squeezing coupling parameter *σ*_0_ in a scenario of practical interest, we consider a circular hole of radius *a* and length *L* extending along the *z* direction and drilled in a homogeneous metallic slab of permittivity *ϵ*(*ω*), with the electron traveling in vacuum parallel to the hole axis. For *ω*_0_ *a*/*c* ≪ 1, we can neglect retardation in the description of the mode, which for an azimuthal number *m*, has an associated electric potential inside the hole (*R* < *a*) of the form $\phi_{0}\left(r\right)=AI_{m}\left(q_{z}R\right)e^{iq_{z}z+im\varphi }$ , and $\phi_{0}\left(r\right)=BK_{m}\left(q_{z}R\right)e^{iq_{z}z+im\varphi }$ in the material region (*R* > *a*). These expressions are given in cylindrical coordinates **r** = (*R*, *φ*, *z*) and involve the modified Bessel functions *K*_*m*_ and *I*_*m*_. Also, the constants *A* and *B* must satisfy the condition $B/A=I_{m}\left(q_{z}a\right)/K_{m}\left(q_{z}a\right)=\epsilon \left(\omega \right)I_{m}^{\prime }\left(q_{z}a\right)/K_{m}^{\prime }\left(q_{z}a\right)$ to guarantee the continuity of the potential and the normal electric displacement at the surface of the hole. We then calculate the corresponding electric field ${\vec{E}}_{0}\left(r\right)=-\nabla \phi_{0}\left(r\right)$ and normalize the field as indicated in Section 2.1.2.

As an example, we take the electron to be focused at the hole center (**R** = 0, approaching with an azimuthal angle *φ*). Since excitations of symmetries ±*m* are degenerate, we can choose the mode as any linear combination of these two. In particular, we take *m* = ±1 waves combined with a relative phase *ψ*, such that the mode field becomes ${\vec{E}}_{0}^{m=\pm 1}\left(R=0,z\right)=\left(-E_{0}/2^{3/2}\right)\left[\hat{R}\left(1+e^{i\left(\psi -2\varphi \right)}\right)+i\;\hat{\varphi }\left(1-e^{i\left(\psi -2\varphi \right)}\right)\right]\;e^{iq_{z}z+i\varphi }$ , with *E*_0_ = *q*_*z*_ *A* acting as a normalization constant. We estimate *E*_0_ from the normalization condition $\int d^{2}R\;|{\vec{E}}_{0}\left(R\right){|}^{2}=2\pi \hbar \omega_{0}/L$ , neglecting the field inside the metal, which should be small because of the condition |*ϵ*(*ω*_0_)| ≫ 1 required to have strong confinement. We obtain $E_{0}^{2}=\hbar \omega_{0}/La^{2}I\left(\eta \right)$ , where *η* = *ω*_0_ *a*/*v* and $I\left(\eta \right)=\int_{0}^{1}x\;dx\;\left\{{\left[I_{0}\left(x\eta \right)+I_{2}\left(x\eta \right)\right]}^{2}/4+I_{1}^{2}\left(x\eta \right)\left(1/x^{2}\eta^{2}+1\right)\right\}$ . Plugging the field into the expressions given above for *k* and *σ*_0_, we obtain the result shown at the end of Section 2.1.2.

### 4.3 Proof of Eq. (9)

We start by considering the coefficient $F_{\ell }^{n}=\left\langle n|\;\hat{S}\left(\sigma_{0}\right)\;e^{-\text{i}\lambda \left(a^{†}a+aa^{†}\right)/2}\times \hat{D}\left({\tilde{\beta }}_{0}\right)\;|n+\ell \right\rangle$ under the following assumptions: (i) high bosonic population in the cavity, implying the condition *n* + *ℓ* ≫ 1, and (ii) small intrinsic electron–cavity couplings |*σ*_0_| ≪ 1 and $|{\tilde{\beta }}_{0}|\ll 1$ , yielding the condition *ℓ*/*n* ≪ 1. We use the ordering theorems mentioned in Methods, Section 4.1, together with the approximation (ii) to write $F_{\ell }^{n}\approx e^{-i\lambda /2}\left\langle n|e^{-a^{†}a^{†}\sigma_{0}^{*}/2}e^{aa\sigma_{0}/2}e^{-i\lambda a^{†}a}e^{{\tilde{\beta }}_{0}^{*}a^{†}}e^{-{\tilde{\beta }}_{0}a}|n+\ell \right\rangle$ . Then, we expand the exponential operators in Taylor series to compute the corresponding matrix element, which leads to the result

(19) $$\begin{array}{ll} F_{\ell }^{n} & \approx e^{-i\lambda \left(n+\ell +\frac{1}{2}\right)}{\overset{\infty }{\underset{i,i^{\prime },j=0}{\sum }}}^{\prime }\;\frac{{\left(-1\right)}^{j+\left(3i^{\prime }-\ell -i\right)/2}}{\left[j+\left(i^{\prime }-\ell -i\right)/2\right]!j!i!i^{\prime }!}\\ & \;\times {\left(\frac{\left|\sigma_{0}\right|}{2}\right)}^{2j+\left(i^{\prime }-\ell -i\right)/2}|{\tilde{\beta }}_{0}{|}^{i+i^{\prime }}\exp\left\{i\left(i-i^{\prime }+\ell \right)\right.\\ & \;\left.\times \arg\left\{\sigma_{0}\right\}/2+i\left(i^{\prime }-i\right)\left(\arg\left\{{\tilde{\beta }}_{0}\right\}+\lambda \right)\right\}\\ & \;\times \frac{\left(n+\ell -i^{\prime }+i\right)!\sqrt{\left(n+\ell \right)!n!}}{\left(n+\ell -i^{\prime }\right)!\left(n+\ell -i^{\prime }+i-2j\right)!}, \end{array}$$

where the prime in the summation symbol indicates that the indices are restricted by the inequalities *n* + *ℓ* − *i*′ + *i* − 2*j* ≥ 0, *n* + *ℓ* − *i*′ ≥ 0, and *j* + (*i*′ − *i* − *ℓ*)/2 ≥ 0, as well as by the condition that *i*′ − *ℓ* − *i* is an even number. In virtue of assumption (ii), the first two inequalities are automatically satisfied. Also, this assumption allows us to follow a procedure similar to the one described in the Methods section of Ref. [20], consisting in using the Striling formula to approximate the factorials and neglecting all the indices in front of *n* when they are not exponentiated. Then, the entire factor in the fourth line of Eq. (19) reduces to (*n* + *ℓ*)^*i*′+2*j*−*ℓ*/2^, and by making the substitutions *m *= (*i*′ − *i *− *ℓ*)/2 and *s* = *j* + *m*, we obtain

(20) $$\begin{array}{ll} F_{\ell }^{n} & \approx e^{i\ell \arg\left\{-{\tilde{\beta }}_{0}\right\}-i\lambda \left(m+\frac{1}{2}\right)}{\overset{\infty }{\underset{i=0}{\sum }}}^{\prime }\;\overset{\infty }{\underset{\begin{array}{c} m=-\left(i+\ell \right)/2\\ s=\max\left\{m,0\right\} \end{array}}{\sum }}{\left(-1\right)}^{i+s}\\ & \;\times {\left(\frac{\left|\sigma_{0}\right|}{2}\right)}^{2s-m}|{\tilde{\beta }}_{0}{|}^{2\left(i+m\right)+\ell }\frac{{\left(n+\ell \right)}^{2s+i+\ell /2}}{s!\left(s-m\right)!i!\left(2m+i+\ell \right)!}, \end{array}$$

where now the prime indicates that the leftmost sum is restricted to even values of *i* + *ℓ*. Finally, by pushing the lower limits of the *m* and *s* sums to *m* = −∞ and *s* = 0, and further using the series expansion of the Bessel functions [55] $J_{\ell }\left(x\right)=\sum_{m=0}^{\infty }{\left(-1\right)}^{m}{\left(x/2\right)}^{2m+\ell }/\left(m+\ell \right)!m!$ , we find that Eq. (20) directly reduces to Eq. (12).

## References

[1] Walls D. F. (1983). Squeezed states of light. Nature.

[2] Gottesman D., Kitaev A., Preskill J. (2001). Encoding a qubit in an oscillator. *Phys. Rev. A*.

[3] Menicucci N. C. (2014). Fault-tolerant measurement-based quantum computing with continuous-variable cluster states. Phys. Rev. Lett..

[4] Slusher R. E., Hollberg L. W., Yurke B., Mertz J. C., Valley J. F. (1985). Observation of squeezed states generated by four-wave mixing in an optical cavity. Phys. Rev. Lett..

[5] Wu L. A., Kimble H. J., Hall J. L., Wu H. (1986). Generation of squeezed states by parametric down conversion. Phys. Rev. Lett..

[6] Bendaña X. M., Polman A., García de Abajo F. J. (2011). Single-photon generation by electron beams. Nano Lett..

[7] Feist A., Huang G., Arend G. Cavity-mediated electron-photon pairs. ..

[8] García de Abajo F. J. (2010). Optical excitations in electron microscopy. Rev. Mod. Phys..

[9] Becker W., Scully M. O., Zubairy M. S. (1982). Generation of squeezed coherent states via a free-electron laser. Phys. Rev. Lett..

[10] Gjaja I., Bhattacharjee A. (1987). Generation of squeezed radiation from a free-electron laser. Phys. Rev. A.

[11] Gjaja I., Bhattacharjee A. (1991). Effective density matrix for free-electron-laser radiation. Phys. Rev. A.

[12] Lobastov V. A., Srinivasan R., Zewail A. H. (2005). Four-dimensional ultrafast electron microscopy. Proc. Natl. Acad. Sci..

[13] Barwick B., Zewail A. H. (2015). Photonics and plasmonics in 4D ultrafast electron microscopy. ACS Photonics.

[14] Aseyev S. A., Ryabov E. A., Mironov B. N., Ischenko A. A. (2020). The development of ultrafast electron microscopy. Crystals.

[15] Barwick B., Flannigan D. J., Zewail A. H. (2009). Photon-induced near-field electron microscopy. Nature.

[16] Feist A., Echternkamp K. E., Schauss J., Yalunin S. V., Schäfer S., Ropers C. (2015). Quantum coherent optical phase modulation in an ultrafast transmission electron microscope. Nature.

[17] Priebe K. E., Rathje C., Yalunin S. V. (2017). Attosecond electron pulse trains and quantum state reconstruction in ultrafast transmission electron microscopy. Nat. Photon..

[18] Morimoto Y., Baum P. (2018). Diffraction and microscopy with attosecond electron pulse trains. Nat. Phys..

[19] Baum P. (2017). Quantum dynamics of attosecond electron pulse compression. J. Appl. Phys..

[20] Di Giulio V., García de Abajo F. J. (2020). Free-electron shaping using quantum light. Optica.

[21] Di Giulio V., Kociak M., García de Abajo F. J. (2019). Probing quantum optical excitations with fast electrons. Optica.

[22] Hayun A. B., Reinhardt O., Nemirovsky J., Karnieli A., Rivera N., Kaminer I. (2021). Shaping quantum photonic states using free electrons. *Sci. Adv.*.

[23] Dahan R., Baranes G., Gorlach A., Ruimy R., Rivera N., Kaminer I. Creation of optical cat and GKP states using shaped free electrons. ..

[24] Rocca M. (1995). Low-energy EELS investigation of surface electronic excitations on metals. Surf. Sci. Rep..

[25] Nagao T., Yaginuma S., Inaoka T., Sakurai T. (2006). One-dimensional plasmon in an atomic-scale metal wire. Phys. Rev. Lett..

[26] Talebi N. (2020). Strong interaction of slow electrons with near-field light visited from first principles. *Phys. Rev. Lett.*.

[27] Kozák M. (2021). Electron vortex beam generation via chiral light-induced inelastic ponderomotive scattering. ACS Photonics.

[28] Talebi N., Bezinová I. (2021). Exchange-mediated mutual correlations and dephasing in free-electrons and light interactions. New J. Phys..

[29] Yuen H. P. (1976). Two-photon coherent states of the radiation field. Phys. Rev. A.

[30] García de Abajo F. J., Dias E. J. C., Di Giulio V. (2022). Complete excitation of discrete quantum systems by single free electrons. *Phys. Rev. Lett.*.

[31] Di Giulio V., García de Abajo F. J. (2020). Electron diffraction by vacuum fluctuations. New J. Phys..

[32] Reid W. T. (1972). Ricatti Differential Equations.

[33] Bishop R. F., Vourdas A. (1986). Generalised coherent states and Bogoliubov transformations. J. Phys. A: Math. Gen..

[34] Glauber R. J., Lewenstein M. (1991). Quantum optics of dielectric media. Phys. Rev. A.

[35] Kfir O. (2019). Entanglements of electrons and cavity photons in the strong-coupling regime. Phys. Rev. Lett..

[36] Yoon J. H., Selbach F., Schumacher L., Jose J., Schlücker S. (2019). Surface plasmon coupling in dimers of gold nanoparticles: experiment and theory for ideal (spherical) and nonideal (faceted) building blocks. ACS Photonics.

[37] Huang Y., Ma L., Li J., Zhang Z. (2017). Nanoparticle-on-mirror cavity modes for huge and/or tunable plasmonic field enhancement. Nanotechnology.

[38] Di Giulio V., Kfir O., Ropers C., García de Abajo F. J. (2021). Modulation of cathodoluminescence emission by interference with external light. ACS Nano.

[39] Kfir O., Di Giulio V., García de Abajo F. J., Ropers C. (2021). Optical coherence transfer mediated by free electrons. *Sci. Adv.*.

[40] Nielsen M. A., Chuang I. L. (2004). *Quantum Computation and Quantum Information (Cambridge Series on Information and the Natural Sciences)*.

[41] Zhao Z., Sun X. Q., Fan S. (2021). Quantum entanglement and modulation enhancement of free-electron--bound-electron interaction. Phys. Rev. Lett..

[42] Yalunin S. V., Feist A., Ropers C. (2021). Tailored high-contrast attosecond electron pulses for coherent excitation and scattering. Phys. Rev. Res..

[43] Bourassa I. T. J. E., Menicucci N. C., Sabapathy K. K. (2020). Progress towards practical qubit computation using approximate Gottesman-Kitaev-Preskill codes. *Phys. Rev. A*.

[44] Guo X., Li N., Yang X. (2022). Approaching the Purcell factor limit with whispering-gallery hyperbolic phonon polaritons in hBN nanotubes. ..

[45] Echternkamp K. E., Feist A., Schäfer S., Ropers C. (2016). Ramsey-type phase control of free-electron beams. Nat. Phys..

[46] Konečná A., Di Giulio V., Mkhitaryan V., Ropers C., García de Abajo F. J. (2020). Nanoscale nonlinear spectroscopy with electron beams. *ACS Photonics*.

[47] Kfir O., Lourenço-Martins H., Storeck G. (2020). Controlling free electrons with optical whispering-gallery modes. Nature.

[48] Dahan R., Nehemia S., Shentcis M. (2020). Resonant phase-matching between a light wave and a free-electron wavefunction. Nature Phys..

[49] Müller N., Hock V., Koch H., Bach N., Rathje C., Schäfer S. (2021). Broadband coupling of fast electrons to high-Q whispering-gallery mode resonators. ACS Photonics.

[50] Huang G., Engelsen N. J., Kfir O., Ropers C., Kippenberg T. J. (2022). Coupling ideality of free electrons with photonic integrated waveguides. ..

[51] García de Abajo F. J., Konečná A. (2021). Optical modulation of electron beams in free space. Phys. Rev. Lett..

[52] Sakurai J. J. (1994). *Modern Quantum Mechanics*.

[53] Chaturvedi S., Sriram M. S., Srinivasan V. (1987). Berry’s phase for coherent states. J. Phys. A: Math. Gen..

[54] Barnett S. M., Radmore P. M. (1997). Methods in Theoretical Quantum Optics.

[55] Gradshteyn I. S., Ryzhik I. M. (2007). Table of Integrals, Series, and Products.

